# Parvalbumin expression does not account for discrete electrophysiological profiles of glutamatergic ventral pallidal subpopulations

**DOI:** 10.1016/j.addicn.2024.100170

**Published:** 2024-08-10

**Authors:** Robert D Graham, Lisa Z Fang, Jessica R Tooley, Vani Kalyanaraman, Mary Christine Stander, Darshan Sapkota, Michelle R Lynch, Joseph D Dougherty, Bryan A Copits, Meaghan C Creed

**Affiliations:** a Department of Anesthesiology, Washington University in St. Louis, United States; b Graduate program in neuroscience, Division of Biological and Biomedical Sciences, United States; c Department of Psychiatry, Washington University in St. Louis, United States; d NINDS Neuroscience Postbaccalaureate Program, Washington University School of Medicine, St. Louis, MO, United States; e Department of Biological Sciences, University of Texas at Dallas, United States; f Department of Genetics, Washington University in St. Louis, United States

**Keywords:** Translating ribosome affinity purification, Ion channels, Electrophysiology, Neuron modeling, Markov chain Monte-Carlo

## Abstract

The ventral pallidum (VP) has emerged as a critical node in the mesolimbic reward system. Modulating the VP can impact the subjective valuation of rewards, reward motivation, and reward seeking under conflict, making it an attractive target for clinical neuromodulation therapies that manage substance use disorders. To understand how to rationally modulate the VP, we need a better understanding of the electrophysiological properties of VP neurons and the molecular and biophysical determinants of these properties. Here, we used patch-clamp electrophysiology to characterize the intrinsic properties of glutamatergic VP (VP_Glu_) neurons and observed two distinct electrophysiological profiles: VP_Glu_ neurons that undergo depolarization block in response to progressively increasing current injection amplitudes and those that were resistant to depolarization block. To explore the mechanisms that could contribute to these distinct profiles, we used targeted ribosome affinity purification to identify ion channel subunits and regulatory proteins by isolating actively transcribed mRNA selectively from VP_Glu_ neurons. We then used this transcriptomic information to implement a Markov Chain Monte Carlo method to parameterize a large population of biophysically distinct multicompartment models of VP_Glu_ neurons conforming to either subpopulation. Based on prior literature suggesting parvalbumin (PV) is expressed in a subset of VPGlu neurons, and that PV expression governs the firing properties of those neurons, we tested the hypothesis that PV expression accounted for differences in subgroups, by increasing the maximal firing frequency and conferring resistance to depolarization block. In contrast, our model determined that PV expression at physiological levels had no effect on maximum firing rate. However, supraphysiological expression levels of PV appeared to induce a depolarization block in previously depolarization block-resistant neuron models, suggesting that other intracellular calcium-binding proteins could play a role in determining the firing phenotype of VP_Glu_ neurons. We corroborated this result with single-cell patch-clamp RT-PCR, which confirmed that PV expression did not distinguish the two electrophysiologically distinct subpopulations. Together, these findings establish that VP_Glu_ neurons are composed of biophysically distinct subpopulations that have not been appreciated in prior studies interrogating the function of this population. With the advent of novel tools for cell-type specific pharmacology and targeted neurostimulation, this understanding will be critical for developing strategies to rationally modulate VP_Glu_ cells to treat disorders characterized by maladaptive reward seeking.

## Introduction

The ventral pallidum (VP) is a critical locus of drug-evoked plasticity, and a promising target for neuromodulation to reduce behavioral symptoms of substance use disorders [1–4]. Addictive drugs induce their rewarding effects through activation of dopaminergic projections to the nucleus accumbens (NAc); this initial release is also critical for drug-induced plasticity that drives long-term changes in behavior in substance use disorders (SUDs) [5–7]. The VP is a primary input and output of both the NAc and ventral tegmental area (VTA) and has emerged as a promising target to manipulate drug sensitization and reinstatement [8–14]. The advent of targeted neuromodulation therapies and cell-type specific pharmacology is reducing the barriers to cell-type specific neuromodulation *in vivo* and holds great promise for improving the translation of preclinical findings into clinical neuromodulation therapies. However, to design rational therapies for SUDs, we need to first understand VP cell types and how they may be leveraged for circuit modulation.

The VP is a highly neurochemically and electrophysiologically heterogeneous nucleus [15,16], and one typical classification of this neuronal heterogeneity is by the primarily expressed neurotransmitter. Specifically, glutamatergic VP (VP_Glu_) neurons are largely non-overlapping with the canonical GABAergic and cholinergic VP neuronal populations [17,18]. Recent work suggests that the balance of activity between GABAergic and VP_Glu_ neurons mediates reward seeking under conditions of conflict, with VP_Glu_ neurons specifically constraining reward seeking in the face of negative consequences [10,14,17–20]. VP_Glu_ neurons are activated by both reward omission and punishment [14,19]. When VP_Glu_ neurons are genetically or optogenetically silenced, mice do not reduce reward intake upon introduction of punishment. This inability to appropriately inhibit reward seeking in the face of aversive consequences is a cardinal feature of SUDs and other compulsive disorders. Thus, neuromodulatory interventions that upregulate activity of these VP_Glu_ neurons could have therapeutic potential for treating disorders characterized by maladaptive reward seeking. However, little is known about the biophysical properties governing activity and excitability of VP_Glu_ neurons, preventing the development of rational therapies to modulate these neurons.

We used patch-clamp electrophysiology to characterize the intrinsic electrophysiological properties of VP_Glu_ neurons. This initial electrophysiological data revealed two distinct subtypes of VP_Glu_ neurons: one population that entered depolarization block (DB) in response to high-amplitude current injections and one that did not enter depolarization block over our range of current injections (NDB). Prior work has established that a subset of VP_Glu_ neurons co-express the calcium-binding protein parvalbumin (PV [17,18,21]). PV rapidly binds to and buffers intracellular calcium, attenuating the Ca^2+^-activated potassium conductance responsible for after hyperpolarization, thereby allowing neurons to fire with lower inter-spike intervals [22–24]. Thus, we hypothesized that the expression of PV accounts for the difference in biophysical properties between the two subpopulations we observed.

To test this hypothesis, we combined patch-clamp electrophysiology, transcriptomics, and computational modeling to parameterize biophysically plausible populations of VP_Glu_ neuron models. Our transcriptomic data revealed the voltage-gated ion channels expressed in VP_Glu_ neurons, which we used to parametrize populations of biophysically distinct DB and NDB VP_Glu_ neurons. We used these model populations to test the hypothesis that calcium sequestration via parvalbumin (PV) accounts for differences between DB and NDB VP_Glu_ neurons. Our modeling argued against this hypothesis, which we corroborated with single-cell RT-PCR, bolstering the utility of our model. These results have generated the substrate for future experimental investigations into mechanisms that may ultimately be leveraged to modulate VP_Glu_ neurons.

## Methods

### Animals

Adult mice were used for patch-clamp electrophysiology and RNA sequencing experiments. We used vGluT2-Cre mice (Slc17a6tm2(cre) Lowl/J, JAX:016,963) crossed with either the Ai14 reporter (B6.Cg-Gt (ROSA)26Sor tm14(CAG-tdTomato)Hze /J, JAX: 007,914) for electrophysiology or to RiboTag strain for Translating ribosome affinity purification (TRAP) experiments (B6J.129(Cg)-Rpl22tm1.1Psam/SjJ, JAX: 029,977). Animals were maintained with ad libitum lab chow and water, on a standard light cycle (ON at 7am, OFF at 7pm). Mice were between 8 and 20 weeks at the time of patch clamp recordings. All procedures were approved by the Institutional Animal Care and Use Committee at Washington University in St. Louis.

### Fluorescent in situ hybridization (FISH)

8-week old vGluT2-Cre mice (*n* = 1F, 2 M) were anesthetized with isoflurane, sacrificed, and brains were rapidly dissected then flash frozen in isopentane at −80 °C. 10 μm sections spanning the rostrocaudal extent of the VP were prepared on a cryostat (Leica CM1850; Leica Biosystems, Deer Park, USA) and collected on Superfrost Plus microscope slides (Fisher Scientific, Pittsburgh, USA). Probes for *Slc17a6* (vGluT2) and *Cre* mRNA were hybridized and fluorescently labeled using the RNAScope Multiplex Fluorescent V2 Assay Kit (Advanced Cell Diagnostics, Newark, USA) according to manufacturer’s instructions. DAPI was used as a counterstain and slides were coverslipped using Gold Prolong antifade mounting medium (Invitrogen, Eugene, OR). Z-stacks encompassing the entire VP were acquired using a confocal microscope (Yogokawa CSU-W1 SoRa Spinning Disk Confocal; Nikon Instruments, Melville, USA) under the 20X objective. Tiff files were then imported into Fiji (v2.15.1) and 300 μm x 300 μm regions of interest (ROIs) were placed within the VP. Cells expressing *Slc17a6* or *Cre* were manually counted for each ROI, and colocalization was determined by coincident detection of both probes within the same nucleus as defined by DAPI. Two ROIs were analyzed per slice, per animal.

### Slice electrophysiology

Whole-cell patch-clamp recordings were made from coronal slices (210 μm) of the VP from adult Ai14 x vGluT2-Cre mice (8F/7 M). Slices were prepared using a vibratome (Leica VT 2100) in ice-cold cutting solution (composition in mM: 0.5 CaCl_2_, 110 C_5_H_14_CINO, 25 C_6_H_12_O_6_, 25 NaHCO_3_, 7 MgCl_2_, 11.6 C_6_H_8_O_6_, 3.1 C_3_H_3_NaO_3_, 2.5 KCl and 1.25 NaH_2_PO_4_) and bubbled continuously with 95 % O_2_ and 5 % CO_2_. Slices were incubated at 32 °C for 30 min in artificial cerebrospinal fluid (aCSF; composition in mM: 119 NaCl, 2.5 KCl, 1.3 MgCl_2_, 2.5 CaCl_2_, 1.0 Na_2_HPO_4_, 26.2 NaHCO_3_, and 11 glucose). This was followed by storage at room temperature until electrophysiological recordings were conducted. Slices were hemisected and superfused with aCSF at 30 ± 2 °C. Borosilicate glass pipettes (5–7MΩ resistance) pulled on a micropipette puller (Narishige PC-100) and filled with a K-gluconate-based solution (composition in mM: 130 K-gluconate, 10 creatin phosphate, 4 MgCl_2_, 3.4 Na_2_ATP, 0.1 Na3GTP, 1.1 EGTA, and 5 HEPES) were used to record neurons. Recordings were made in current clamp. Negative current was injected into the cell and the resulting voltage change was averaged and used to measure input resistance. Resting membrane potential was calculated from these recordings during the 0 pA injection portion of the recording. Responses to successive current injections were measured from 0 to 140 pA in steps of 10 pA for 500 ms. Afterhyperpolarization was measured at rheobase current injection and was calculated as the difference between the action potential threshold and the peak negative voltage of the AHP. Signals were amplified, filtered at 2 kHz, and digitized at 10 kHz using a MultiClamp 700B amplifier and Digidata 1550 (Molecular Devices). Clampex version 11.4 (Molecular Devices) was used for data acquisition and analysis of electrophysiological parameters. Neurons were visualized using an Olympus x560 upright microscope; field LED illumination (CoolLED) was used to visualize cells labeled with tdTomato (560 nm). All agents were purchased from Sigma Biosciences.

### Slice electrophysiology and single cell RT-PCR

The cytoplasm of single vGluT2+ cells was collected after electrophysiological recording in the VP of Ai14 x vGluT2-Cre mice (4F/4 M). Patch-clamp electrophysiology was performed as above, apart from using an internal solution (composition in mM: 105 K-gluconate, 30 KCl, 10 phosphocreatine, 4 ATP-Mg, 0.3 EGTA, 0.3 GTP-Na, 10 HEPES) made under RNase-free conditions. After completion of the recording, the cytoplasm was aspirated with negative pressure, the tip of the patch pipette was broken, and the cellular contents expelled into an RNase-free tube and stored at −80 °C until all cells were collected before proceeding to library preparation in parallel. cDNA was synthesized from cellular RNA using SuperScript III reverse transcriptase (Thermo Fisher Scientific, Waltham, MA) according to manufacturer’s instructions. cDNA first underwent a pre-amplification step using 100 nM primers as follows: 94 °C for 2 min, then 20 cycles of 94 °C for 30 s, 61 °C for 30 s, and 72 °C for 35 s, finally followed by 72 °C for 5 min. A second PCR reaction was then performed using 2 uL of the pre-amplification product as the template, and 0.5 – 1 uM of the same primers under the conditions reported above for 35 cycles. The final PCR products were run on 2 % agarose gels and visualized using SYBR Green (Lonza, Basel, Switzerland) in a UVP GelSolo imager (Analytik Jena). A positive control (bulk VP tissue) and a no template control were run alongside all single cell samples.

Specific primers for mouse *Pvalb* (target gene) and *18S* (the small ribosomal subunit and positive control) were synthesized based on previous reports or designed based on published sequences: *Pvalb*: Sense, GCAGACTCCTTCGACCACAA; Antisense, TCAGAATGGACCCCAGCTCA. *18S:* Sense, CTCAACACGGGAAACCTCAC; Antisense, CGCTCCACCAA CTAAGAACG

### RNA sequencing following translating ribosome affinity purification (TRAP)

Adult vGluT2-Cre x RiboTag mice (9–14 weeks of age) were group housed prior to tissue extraction. Each sample contained pooled tissue from 7 to 11 mice, males and females were included in each sample and 5 independent samples were sequenced for a total of 43 mice (21 M / 24 F). Mice were deeply anesthestized with isoflurane, brains were extracted and a coronal slab containing the VP was dissected using a custom 3D printed brain matrix tailored for dissecting the VP between AP +0.7 mm and −0.2 mm. Coronal slabs were then transferred to a sterile petri-dish treated with RNAse-X on dry ice, containing homogenization buffer and the VP was microdissected using a reusable 0.35 mm biopsy punch. Punches were taken with deliberate care to avoid the neighboring substantia innominata, lateral hypothalamus and bed nucleus of the striatum terminalis. As a result, samples may have been contaminated by neighboring striatum above the anterior commissure or from the nucleus accumbens. However, these areas do not express vGluT2, and would not contaminate our samples precipitated from ribosomes in Cre-positive cells. VP punches were transferred into 2 mL ice cold homogenization buffer (20 mM HEPES (pH 7.3), 150 mM KCl, 10 mM MgCl_2_, 0.5 mM dithiothreitol, 100 μg/mL cycloheximide, EDTA-free protease inhibitor cocktail (Roche), 5 μL/mL RNasin and 2 μL/mL Superasin). Each sample was homogenized with a motor-driven Teflon glass homogenizer and centrifuged for 10 min at 20,000 g, 4 °C. NP-40 and 1, 2-diheptanoyl-sn-glycero-3-phosphocholine were added to the supernatant at final concentrations of 1% and 30 mM, respectively. After incubation on ice for 5 min, the lysate was centrifuged for 10 min at 13,000 g, 200 μL of the sample was removed and 500 μL of Trizolwas added before storage at −80 °C to constitute the Input fraction. Streptavidin Dynabeads (300 μL, Invitrogen Cat#65601) pre-conjugated with Protein L (ThermoFischer Cat# 29997) and Anti-HA antibody (Biolegend Cat# 901,513) in homogenization buffer were added to the remaining 1800 μL of the sample [25]. Beads were collected on a magnet at 4 °C and washed for 5 cycles with 1 ml 350 mM KCl buffer before resuspension in 700 μL of Trizol. After 5 min at room temperature, samples were stored at −80 °C before RNA isolation.

RNA from the input and TRAP fraction for all samples was isolated following the Zymo RNA Clean & Concentrator 5 protocol with oncolumn DNase digestion. RNA integrity was assayed using 1 μL / sample on an RNA Pico chip on a Bioanalyzer 2100 and only samples with RIN > 7.9 were considered for subsequent analysis. TRAP and unbound fraction were sent to the Washington University Genome Technology Access Center (GTAC) to produce libraries for sequencing by NextSeq 500 (Illumina) with high-output single read sequencing for 75 cycles. Samples were prepared according to library kit manufacturer’s protocol, indexed, pooled, and sequenced on an Illumina NovoSeq. Basecalls and demultiplexing were performed with Illumina’s bcl2fastq software and a custom Python demultiplexing program with a maximum of one mismatch in the indexing read. RNA-seq reads were then aligned to the Ensembl release 76 primary assembly with STAR version 2.5.1a [26]. Gene counts were derived from the number of uniquely aligned unambiguous reads by Subread:featureCount version 1.4.6-p5 [27]. Isoform expression of known Ensembl transcripts were estimated with Salmon version 0.8.2 [28]. Sequencing performance was assessed for the total number of aligned reads, total number of uniquely aligned reads, and features detected. The ribosomal fraction, known junction saturation, and read distribution over known gene models were quantified with RSeQC version 2.6.2 [29].

All gene counts were then imported into the R/Bioconductor package EdgeR [30] and TMM normalization size factors were calculated to adjust for samples for differences in library size. Ribosomal genes and genes not expressed in the smallest group size minus one samples greater than one count-per-million were excluded from further analysis. The TMM size factors and the matrix of counts were then imported into the R/Bioconductor package Limma [31]. Weighted likelihoods based on the observed mean-variance relationship of every gene and sample were then calculated for all samples with the voom With Quality Weights [32]. The performance of all genes was assessed with plots of the residual standard deviation of every gene to their average log-count with a robustly fitted trend line of the residuals. Differential expression analysis was then performed to analyze for differences between the TRAP-isolated and INPUT fraction and the results were filtered for only those genes with Benjamini-Hochberg false-discovery rate adjusted p-values < 0.05. Data are available at the Gene Expression Omnibus under accession number: GSE272940.

### Multicompartment VP_Glu_ neuron model

We developed a multicompartment cable model of a VP_Glu_ neuron using the NEURON simulation environment (v8.2 [33]) within the Python programming language [34]. Presently, the morphology of VPGlu neurons is uncharacterized. Therefore, we implemented a simplified ball-and-stick morphology that is commonly used to study neurons in the central nervous system while affording computational simplicity [35–37]. We modeled the somata with a length and diameter of 10 μm and an axon hillock with a length of 30 μm and diameter of 1 μm [36,37]. We modeled the dendrite as a single branch with a diameter of 0.3 μm and a length of 1371 μm to better reproduce the experimentally measured input resistances of VP_Glu_ cells. We modeled a myelinated axon composed of 100 nodes of Ranvier and 99 internodal regions as described previously [38].

We included explicit representations of ion channels found in VP_Glu_ cells revealed by our TRAP transcriptomic counts (Fig 2d). Specifically, we included models of the voltage-gated sodium channel Nav1.6 [39], a delayed-rectifier potassium channel [40], an A-type potassium channel [40], a small-conductance calcium-activated potassium channel (i.e., SK [41]), a large-conductance calcium- and voltage-activated potassium channel (i.e., BK [42]), an M-type potassium current [43], an l-type calcium channel [44], and a T-type calcium channel [44]. In some simulations, where noted below, we extended the model of calcium dynamics to account for buffering produced by the calcium-binding protein parvalbumin [45].

To reduce dimensionality and associated computational demand during model parametrization, we expressed these channels only in the axon hillock and modeled the somatic and dendritic compartments as passive cables with only a linear leak conductance and membrane capacitance [46]. The 100 nodal compartments of the axon contained NEURON’s built-in model of Hodgkin-Huxley nodal dynamics (i.e., the ‘hh’ mechanism in NEURON) to facilitate action potential propagation along the axon, while the myelinated internodal regions were modeled as passive cables (i.e., contained only a linear leak conductance and a membrane capacitance [38,46];.

### Parametrizing the VP_Glu_ neuron model population

To generate a population of biophysically distinct VP_Glu_ neuron models with variable ion channel expression profiles, we implemented Goodman and Weare’s Affine-Invariant Markov Chain Monte Carlo method (MCMC [47]) using the *emcee* Python package (Fig 2d,e). MCMC methods have been previously used in multicompartment cable modeling to simultaneously estimate the values of several ionic current parameters [48,49], and to generate *de novo* combinations of maximal ion channel conductances to model biophysically distinct neuron populations [46,50]. We employed the latter approach to generate populations of biophysically distinct VP_Glu_ DB and NDB neuron models that reproduce experimentally measured electrophysiological characteristics of VP_Glu_ DB and NDB neurons. MCMC methods use Bayes’ theorem to estimate the posterior probabilities of a given combination of parameter values describing a system based on experimental data and user-defined priors. We assumed uniform distributions for each parameter as priors and constrained each distribution to physiologic ranges (e.g., an ionic conductance must be greater than or equal to zero). Functionally, our implementation of the MCMC method serves as a method to systematically generate and test hundreds of thousands of combinations of maximal ion channel conductances and determine which combinations produce VP_Glu_ models whose electrophysiological characteristics resemble those of our experimentally measured neurons (Fig 2f). For each class of VP_Glu_ neuron (i.e., DB and NDB), we ran the MCMC method ten times, with each successive run injecting progressively increasing variance of Gaussian noise in the initial guess for each parameter’s value to ensure the algorithm fully explored each parameter space. Each run of the MCMC algorithm utilized 400 independent Markov chains (i.e., ‘walkers’) which iterated their unique combinations of parameter values 25 times per run.

We evaluated each tested parameter set on its ability to reproduce experimentally measured electrophysiological characteristics (i.e., validation metrics) of either DB or NDB neurons. For each tested parameter set, we calculated its resulting input resistance, F-I curve, threshold in response to a ramp current injection, and square-pulse current injection amplitude which produced the highest firing frequency. For calculating each of these validation metrics, we mimicked the current injection protocols performed in our slice physiology experiments. To calculate a model’s ramp current threshold, we injected a 1-second current ramp that began at a value of 0 pA and increased linearly to 150 pA. To calculate a model’s input resistance, we injected −5 pA of current for 500 ms and divided the resulting change in membrane voltage by the injected current. To calculate a model’s F-I curve, we sequentially injected square current pulses of 500 ms duration between 0 pA and 130 pA with a 10 pA step size between pulses and counted the number of resulting action potentials with peaks above 0 mV. We determined the current injection amplitude which produced the highest firing frequency from the computed F-I curve.

We calculated the normalized distance between each parameter set’s resultant validation metrics and the mean of each experimentally measured value. For comparing model and experimental F-I curves, we calculated the sum of errors between each parameter set’s resultant F-I curve and the mean experimental F-I curve. We averaged the normalized distance across all metrics to calculate a “score” for each parameter set, where a lower score indicates a given parameter set’s validation metrics were closer to the experimentally measured means.

### Simulation details

For all NEURON simulations, we used a temperature of 23 °C, an initialization membrane potential of −60 mV, and an integration time step of 25 μs. We calculated each compartment’s time-varying membrane voltage using a backward Euler implicit integration method. For all simulations, we allowed 500 ms prior to current clamp stimulation to ensure each model’s membrane potential reached its equilibrium resting potential prior to perturbation.

## Results

### Electrophysiological characteristics of VP_Glu_ neurons

**Fig. 1.**
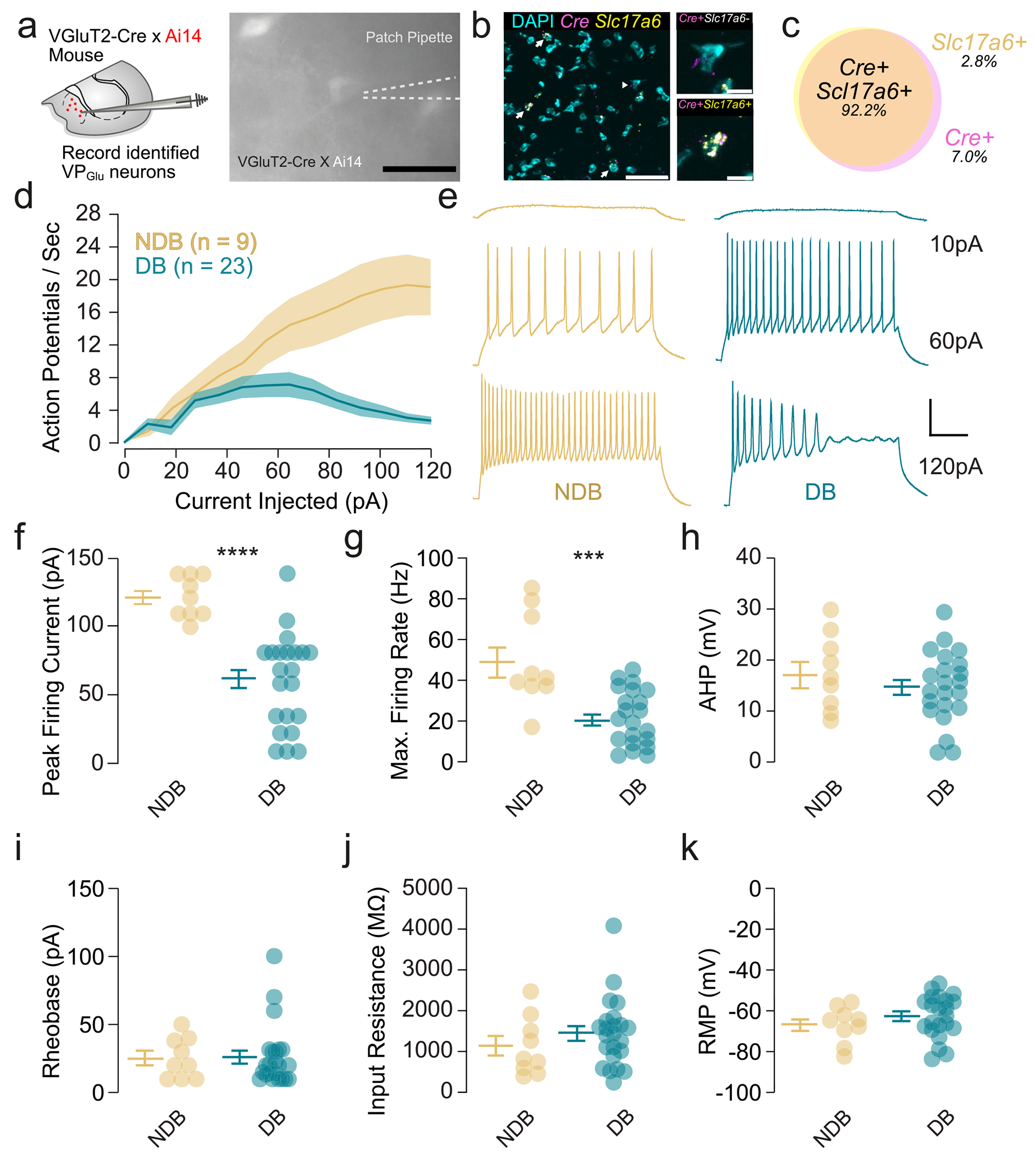
VP_Glu_ neurons exhibit two electrophysiologically distinct profiles. a) Schematic of experiment (scale bar = 45 μm). b) Representative fluorescent in situ hybridization image showing co-localization of *Cre* and *Slc17a6* mRNA (scale bar = 50 μm) in the VP. Insets show representative *Cre*+/*Slc17a6*− cell (top) and *Cre*+/*Slc17a6*+ cell (bottom, scale bar = 10 μm). c) Quantification of *Slc17a6* and *Cre* mRNA found 92.2 % co-localization between the two mRNA species at the single cell level. d) Frequency-current curves of VP_Glu_ neurons that do not experence depolarization block (NDB; 9 cells from 9 mice) and those that do (DB; 23 cells from 13 mice) . Representative traces (DB, blue; NDB, gold) are shown. e) Representative traces ofNDB and DB VP_Glu_ neurons (scale bar = 25 mV, 100 ms). f) The current at which the NDB and DB neurons fired the most action potentials was significantly different (NDB, 122.2 ± 5.212 pA; DB, 53.18 ± 6.493 pA; *t* = 6.418, *p* < 0.0001). g) The NDB VP_Glu_ neurons fire have a significantly higher maximum firing rate as compared to the DB VP_Glu_ neurons (NDB, 48.89 ± 7.624 Hz; DB, 20.76 ± 2.945 Hz; *t* = 4.222, *p* < 0.0002). h-k) No significant differences were found between the two groups when comparing their afterhyperpolarization (NPB, 17.15 ± 2.480 mV; DB, 14.84 ± 1.489; *t* = 0.8200, *p* = 0.4189), resting membrane potential (NDB, −66.88 ± 2.958; DB, −62.64 ± 2.222; *t* = 1.071, *p* = 0.2932), rheobase (NDB, 25.32 ± 4.949 pA; DB, 26.26 ± 4.652 pA; *t* = 0.1160, *p* = 0.9084), and input resistance (NDB, 1124 ± 239 MΩ; DB, 1448 ± 15.8 MΩ; *t* = 1.015, *p* = 0.3182).

We sought to characterize the intrinsic excitability and passive membrane properties of VP_Glu_ neurons, using patch-clamp electrophysiology. We used a transgenic mouse line where a Cre-dependent fluorescent reporter is expressed in vGluT2 cells (vGluT2 Cre x Ai14; Fig 1a–c). We found two distinct populations based primarily on entry into depolarization block and maximum firing rates. We recorded the intrinsic excitability of VP_Glu_ neurons by injecting increasing amounts of current and found over the course of this experiment that two phenotypes emerged: VP_Glu_ neurons that went into depolarization block (DB) and those that did not (NDB) (Fig 1d,e). NDB neurons continued to fire action potentials through the entire current step, even at high current injections, which is highlighted by a frequency-current (F-I) curve (Fig 1d). The current at which DB neurons reached their maximum firing rate was significantly lower than NDB neurons (Fig 1f), and the maximum firing rate of DB neurons was significantly slower as well (Fig 1 g). Additionally, we measured the passive membrane properties of VP_Glu_ neurons, and unlike the intrinsic excitability, no differences in after hyperpolarization, rheobase, input resistance, or resting membrane potential were found between NDB and DB neurons (Fig 1h–k).

**Fig. 2.**
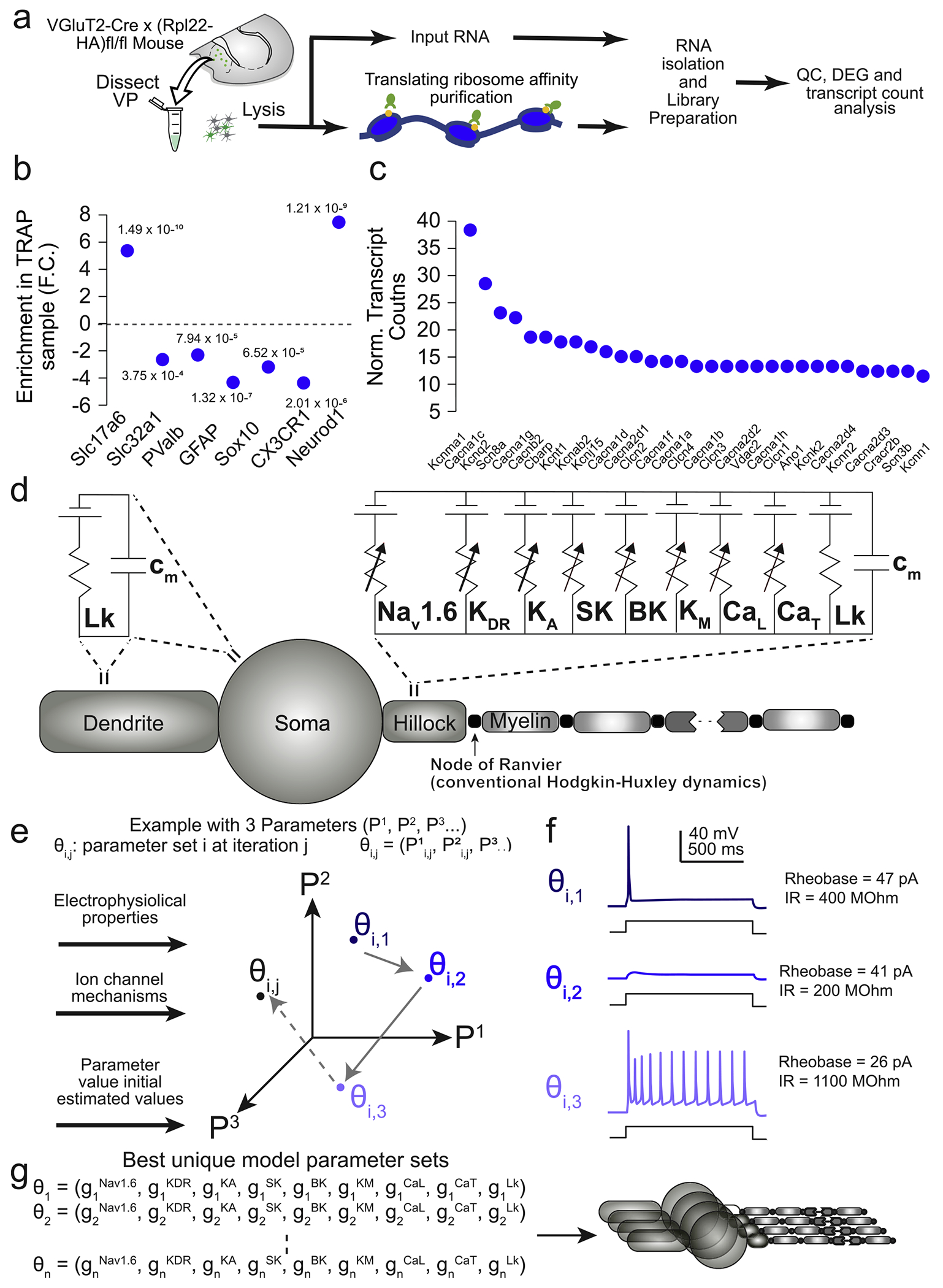
Parameterizing two populations of VP_Glu_ neurons based on RNA sequencing data. a) Experimental schematic of TRAP protocol to isolate actively transcribed RNA from VP_Glu_ cells. ‘ b) Confirmation that vGluT2 (*Slc17a6*) and *NeuroD1* were significantly upregulated in the TRAP fraction relative to input. VGAT (*Slc32a1*), parvalbumin (*Pvalb*) markers of astrocytes (*GFAP*), oligodendrocytes (*Sox10*) and microglia (*CX3CR1*) were all significantly down-regulated in the TRAP fraction relative to input. P values are indicated directly on plot. c) Ion channels were ranked according to highest normalized transcript count in the TRAP fraction for inclusion in subsequent model parameterizations. d) Multicompartment cable model of a VP_Glu_ neuron. We included explicit representations of 8 active ion channels with high normalized transcript counts: Nav1.6, delayed-rectifier potassium, A-type potassium, small-conductance calcium-activated potassium (SK), large-conductance calcium- and voltage-activated potassium (BK), M-type potassium, l-type calcium, and T-type calcium. e) Markov Chain Monte Carlo (MCMC) method for parametrizing populations of DB and NDB model neurons. For visualization purposes, a three-dimensional example with three unknown parameters (P1, P2, and P3) is shown. Our implementation had nine unknown parameters: one for each of the eight active ion channels included in the multicompartment model, and one for the passive leak conductance. f) Example of VP_Glu_ neuron model behavior changing as the MCMC algorithm explores parameter space. g) The output of the MCMC algorithm is a set of parameter combinations (i.e., sets of maximal ion channel conductances) that produce VP_Glu_ DB and NDB neuron model populations that reproduce electrophysiological features of our experimental data (Fig 1). Abbreviations: vGluT2 – vesicular glutamate transporter 2 VGAT – vesicular GABA transporter, See table 1 for gene names of enriched ion channels.

We next used translating ribosome affinity purification (TRAP) to determine which genes encoding ion channel subunits and regulatory proteins are actively translated in VP_Glu_ neurons, to inform biophysically plausible model populations of VP_Glu_ neurons. Briefly, we isolated and sequenced mRNA bound to ribosomes selectively in VP_Glu_ neurons (the TRAP sample) alongside mRNA isolated from the entire VP (the INPUT sample), and normalized transcript counts for each sample. We validated that relative to INPUT, the TRAP sample was significantly enriched in *Slc17a6* (encoding for vGluT2) and Neurod1 (a pan-neuronal marker). Consistent with prior work, we found that *Slc32a1* (encoding for the vesicular GABA transporter; vGAT) and *Pvalb* (encoding for parvalbumin) were more highly expressed in the input fraction relative to the TRAP fraction. Further corroborating the quality and selectivity of our isolation, *Gfap, Sox10* and *Cx3cr1*, which have been used to selectively mark glial cells, oligodendrocytes and microglia respectively [51–54], were also downregulated in our TRAP samples (Fig 2b). We next rank-ordered genes most highly expressed in the TRAP samples after normalizing to total transcript counts, and identified several ion channel subunits and auxiliary proteins that are enriched in the TRAP samples (Table 1). We used these gene counts to guide inclusion of ionic conductances in our neuronal model.

### Model population characteristics

**Fig. 3.**
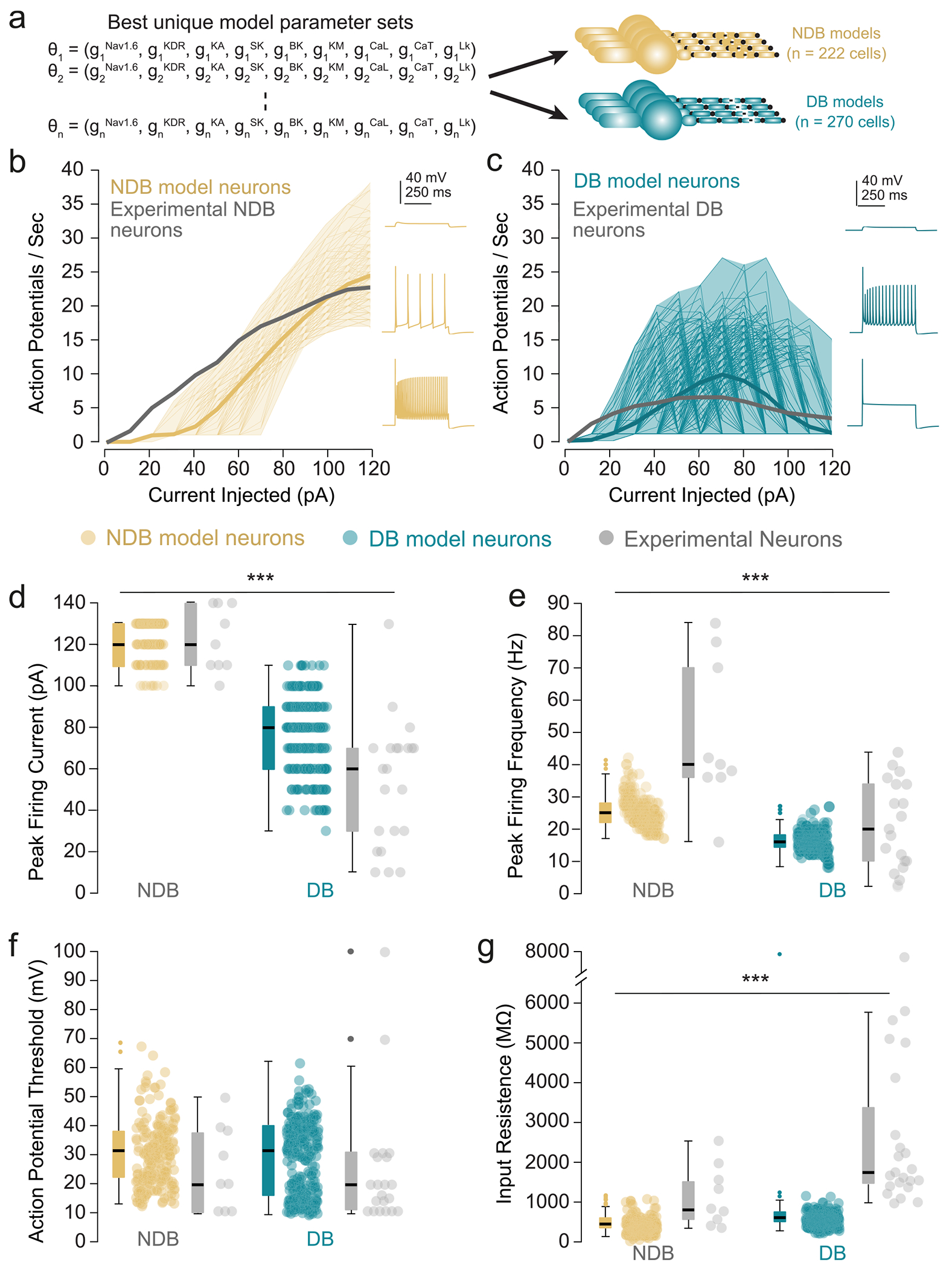
Comparing experimental and multicompartment cable model VP_Glu_ populations. a) The MCMC algorithm produced 222 parameter combinations (i.e., combinations of maximal ion channel conductances) that described NDB VP_Glu_ neuron models, and 270 parameter combinations that described DB VP_Glu_ neuron models. b) Comparing FI-curves between VP_Glu_ NDB model and experimental neurons. The solid gray line represents the mean experimental population FI-curve. The solid gold line represents the mean model population FI-curve. Thin translucent gold lines represent individual model FI-curves, and the gold shaded region represents the range of model population FI-curves. Current clamp responses at three progressively increasing current injection amplitudes from a representative model are shown on the right. c) Comparing FI-curves between VP_Glu_ DB model and experimental neurons. The solid gray line represents the mean experimental population FI-curve. The solid blue line represents the mean model population FI-curve. Thin translucent blue lines represent individual model FI-curves, and the blue shaded region represents the range of model population FI-curves. Current clamp responses at three progressively increasing current injection amplitudes from a representative model are shown on the right. d-g) Comparing distributions of electrophysiological characteristics between VP_Glu_ NDB (gold) and DB (blue) neuron models and their corresponding experimental (gray) distributions. d) Peak firing current (NDB, 121.6 ± 9.5 pA; DB, 76.5 ± 18.4 pA) e) peak firing frequency (NDB, 25.32 ± 4.58 Hz; DB, 15.84 ± 3.04 Hz); f) rheobase (NDB, 31.6 ± 10.9 pA; DB, 29.7 ± 12.6 pA; *U* = 27,555.5, *p* = 0.12); g) input resistance (NDB, 521.2 ± 183.5 MΩ; DB, 624.4 ± 166.0 MΩ). Mann-Whitney *U test* between two model populations; ***p < 0.001 *Abbreviations: MCMC* – *markov chain monte carlo, NDB* – *non-depolarization block, DB* - *depolarization block*.

The MCMC method produced a final population of 222 NDB VP_Glu_ model neurons and 270 DB VP_Glu_ neuron models (Fig 3a). Both the DB and NDB model populations produced similar current-clamp responses (Fig 3b,c) and distributions of electrophysiological properties (i.e., F-I curve, peak firing current, ramp threshold) (Fig 3b–g) to our experimental populations. Our model populations may represent the less-excitable portion of each experimental population because they systematically underestimate the upstroke of the F-I curve (Fig 3b,c) and input resistance (Fig 3 g) of the experimental populations. However, because our model populations capture the majority of the variance in electrophysiological characteristics of their experimental counterparts, we believe our NDB and DB populations provide valuable insight into the biophysical underpinnings of differences between NDB and DB VP_Glu_ neurons.

### Effect of parvalbumin on VP_Glu_ neuron model firing phenotype

We aimed to test the hypothesis that parvalbumin (PV) is a marker of fast-spiking NDB VP_Glu_ neurons. PV is a calcium-binding protein and a common marker for fast-spiking interneurons throughout the brain. Our specific hypothesis was that adding a model of PV-induced calcium buffering to the calcium dynamics of our VP_Glu_ neurons would cause the DB model population’s F-I curve (i.e., an increase in firing rate to an inflection point followed by a decrease with respect to current injection amplitudes) to shift to an NDB-shaped F-I curve (i.e., an approximately linear increase in firing rate in response to increased current injection amplitudes), without affecting the overall shape of the NDB F-I curve.

**Fig. 4.**
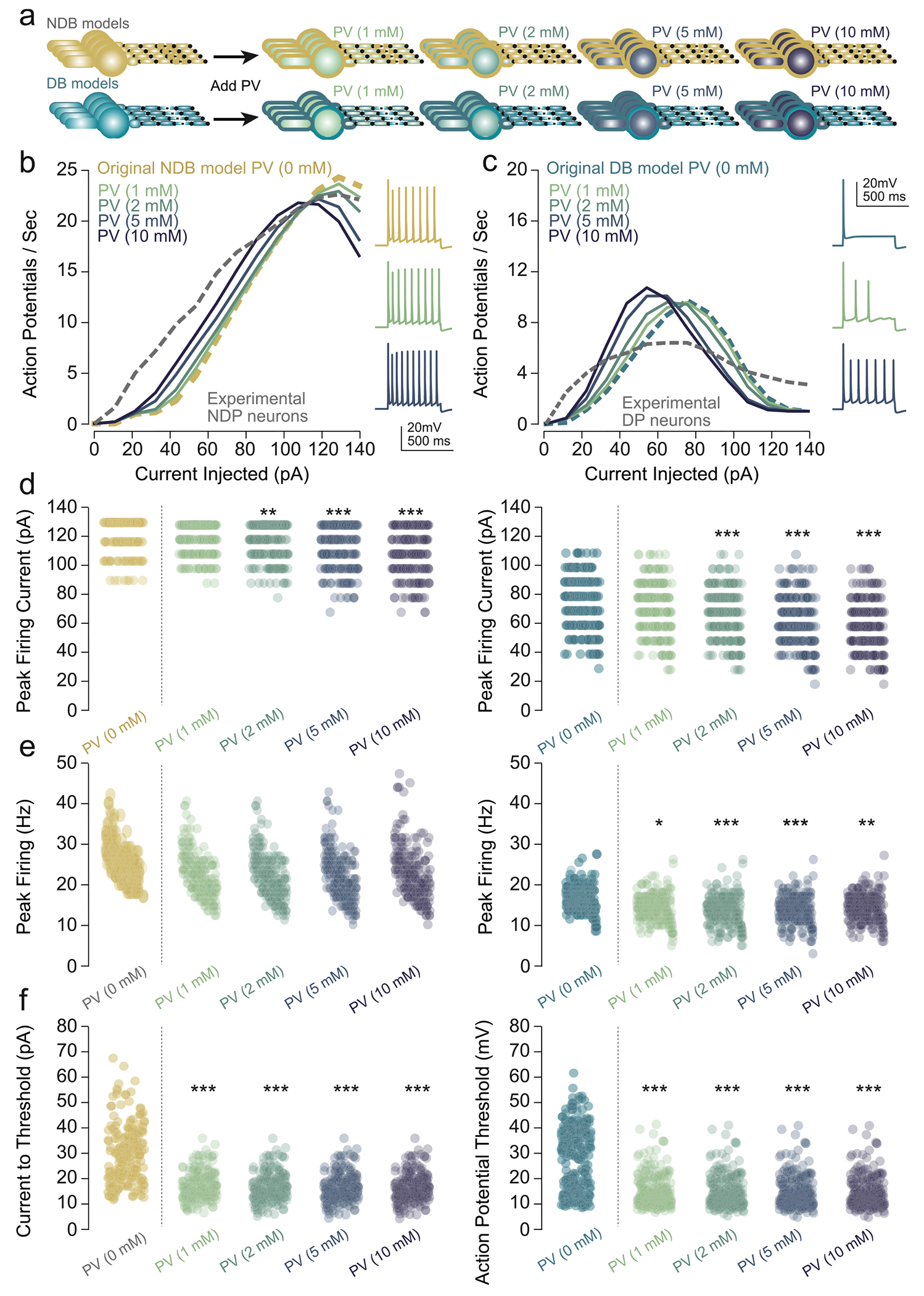
Effect of including parvalbumin (PV) in DB and NDB neuron models a) We simulated the effect of varying initial concentrations of PV (1 mM, 2 mM, 5 mM, and 10 mM) on the firing phenotype of DB and NDB VP_Glu_ neuron models. b) Effect of PV on the FI-curve of NDB models. The dashed gray line indicates the experimental population’s average FI-curve, and the dashed gold line indicates the model population’s average FI-curve without PV. Solid green and purple lines indicate the FI-curves of NDB neurons in response to varying levels of initial PV concentration. Responses of a representative model with three different initial PV concentrations (0, 1, 5 mM) to a 50 pA current injection are shown on the right. c) Effect of PV on the FI-curve of DB models. The dashed gray line indicates the experimental population’s average FI-curve, and the dashed blue line indicates the model population’s average FI-curve without PV. Solid green and purple lines indicate the FI-curves of DB neurons in response to varying levels of initial PV concentration. Responses of a representative model with three different initial PV concentrations (0, 1, 5 mM) to a 50 pA current injection are shown on the right. d-f) Effect of increasing PV initial concentration on the electrophysiological properties of NDB and DB models. Yellow and blue scatter plots indicate the model property value with no PV in NDB and DB models, respectively. Green and purple scatter plots indicate model property value in response to progressively increasing initial PV concentration values. Left column scatter plots indicate property values for NDB models while right column scatter plots indicate values for DB models for d) peak firing current (NDB PV 0mM: 121.6 ± 9.5 pA, 1 mM: 120.0 ± 10.6 pA, 2 mM: 117.6 ± 12.2 pA, 5 mM: 112.9 ± 14.8 pA, 10 mM: 109.7 ± 15.6 pA; DB PV 0 mM: 76.5 ± 18.4 pA, 1 mM: 72.5 ± 17.9 pA, 2 mM: 68.7 ± 16.8 pA, PV 5 mM 62.7 ± 16.9 pA, 10 mM: 59.1 ± 17.0 pA), e) peak firing frequency (NDB PV 0mM: 25.32 ± 4.58 Hz, 1 mM 24.85 ± 4.67 Hz, 2 mM: 24.53 ± 4.92 Hz, 5 mM: 24.46 ± 5.25 Hz, 10 mM: 25.16 ± 5.80 Hz; DB PV 0 mM: 15.84 ± 3.04 Hz, 1 mM: 15.14 ± 3.15 Hz, 2 mM: 14.59 ± 3.40 Hz, 5 mM: 14.75 ± 3.26 Hz, 10 mM 15.29 ± 3.21 Hz), and f) ramp threshold (NDB PV 0mM: 31.6 ± 10.9 pA, 1 mM: 19.0 ± 5.4 pA, 2 mM: 18.5 ± 5.4 pA, 5 mM: 18.3 ± 5.5 pA, 10 mM: 18.3 ± 5.5 pA; DB PV 0 mM: 29.7 ± 12.6 pA, 1 mM: 16.7 ± 6.0 pA, 2 mM: 16.3 ± 6.0 pA, 5 mM: 16.2 ± 6.0 pA, 10 mM: 16.2 ± 6.0 pA. Comparisons are Mann-Whitney U-Test between original model population and model populations with simulated PV concentration, *p < 0.05, **p < 0.01, ***p < 0.001. *Abbreviations: PV* – *parvalbumin, NDB* – *non-depolarization block, DB* - *depolarization block*.

We simulated initial concentrations of PV between 1 and 10 mM in both DB and NDB VP_Glu_ neuron model populations (Fig 4a), and calculated each model population’s average F-I curve in response to each simulated concentration of PV (Fig 4b,c). In both DB and NDB neuron models, increasing initial PV concentration consistently shifted the FI-curve to the left (i.e., increased the overall excitability of the population). In the DB model population, including PV did not significantly affect the overall shape of the FI-curve regardless of initial PV concentration (Fig 4c). Interestingly, in the NDB model population, including PV (particularly at initial concentrations between 2 and 10 mM) pushed the model population mean FI-curve to resemble a DB phenotype (Fig 4b). Progressively increasing PV concentration also produced a slight reduction of peak firing currents (Fig 4d) peak firing frequency (Fig 4e) and ramp threshold (Fig 4f). Counter to our hypothesis, these data establish that simulated PV expression does confer resistance to depolarization block and is thus unlikely to be enriched in the NDB-like phenotype. Instead, simulating PV expression increased the spike frequency for a given current injection, but resulted in entry into depolarization block at lower current injections, suggesting that PV may be associated with the DB-like phenotype of VP_Glu_ neurons.

### Parvalbumin expression in VP_Glu_ subpopulations

**Fig. 5.**
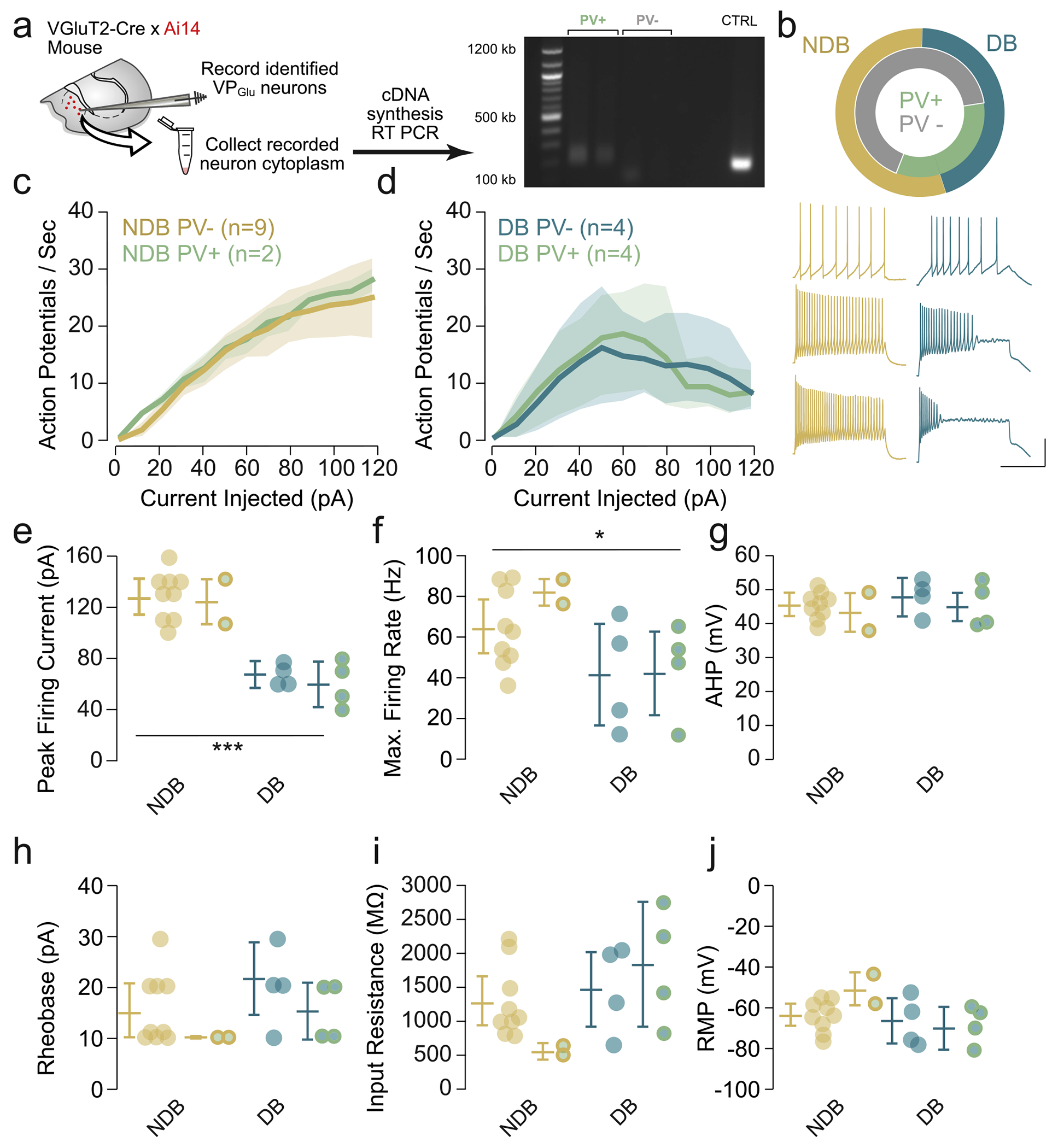
*Pvalb* expression does not account for the electrophysiological differences between VP_Glu_ subpopulations. a) Graphical summary depicting single-cell RT-PCR workflow (left). Cytoplasm from VP_Glu_ collected after whole-cell patch clamp recording was used to synthesize cDNA and RT-PCR was performed. A representative gel image (right) of *Pvalb* expression in VP_Glu_ with PV-positive (PV+) cells in NDB (lane 2) and DB (lane 3) subpopulations, and PV-negative (PV−) cells in NDB (lane 4) and DB (lane 5) subpopulations. No template control (lane 7) and bulk VP positive control (lane 8) are also shown. b) Schematic representation of the proportion of PV+ and PV− cells in VP_Glu_ NDB and DB subpopulations. c-d) F-I curves demonstrating comparable AP firing across a range of injected driving currents between PV+ and PV− cells in both NDB (c) and DB (d) VP_Glu_ subpopulations. Representative traces from *Pvalb*-expressing NDB (gold) and DB (blue) cells after injecting a driving current of 10 pA (top), 60 pA (middle), and 120 pA (bottom). Scale = 500 ms, 40 pA. e) Electrophysiological parameters including peak firing current (e), max firing rate (f), AHP (g), rheobase (h), input resistance (i), and RMP (j) are consistent between PV+ and PV− cells within NDB and DB subpopulations. Differences in peak firing current and max firing rate between NDB and DB subpopulations persist (statistical comparisons between NDB and DB populations only, ***p < 0.001).

To establish whether PV was expressed selectively in either VP_Glu_ subpopulation, we performed whole-cell patch-clamp then extracted the cytoplasm and performed single-cell reverse transcriptase polymerase chain reaction (sc RT-PCR) for *Pvalb* (Fig 5a). We first characterized VP_Glu_ neurons as either NDB or DB, and then confirmed whether the recorded neuron expressed *Pvalb* via RT-PCR. We found that both NDB and DB populations contained PV-positive neurons. Specifically, 2 of 11 NDB cells tested were PV-positive while 4 of 8 DB cells were PV-positive (Fig 5b). Critically, all included neurons were positive for the ribosomal subunit 18S, ruling out the possibility of false negatives due to inadequate cellular aspiration. This proportion of PV-positive neurons is consistent with prior work from our group and others, in which the majority of VP_Glu_ neurons (75–90 %) did not express PV, as measured at the mRNA [17] and protein levels [18].

Within both NDB and DB populations, we classified neurons as PV-positive and PV-negative. The F-I curves were not different between PV-positive and PV-negative neurons within a given subpopulation, as a range of driving currents elicited comparable AP firing between both PV-positive and PV-negative cells (Fig 5c). Similarly, no differences were observed between PV-positive and PV-negative neurons within NDB and DB populations for peak firing current, max firing rate, AHP, rheobase, input resistance, or RMP (Fig 5e–j). Importantly, all NDB cells, regardless of *Pvalb* expression, achieved peak AP firing (Fig 5e) and had a higher maximum firing rate (Fig 5f) in comparison to DB cells. These findings thus corroborate our modeling result (Fig 4), and together confirm that *Pvalb* expression is not a sufficient marker for the electrophysiological phenotype of VP_Glu_ neurons.

## Discussion

The VP has been recognized for decades as a “limbic motor interface” [55]. The VP integrates inputs from the frontal cortex, extended amygdala and NAc and in turn modulates activity of basal ganglia output nuclei in the thalamus and midbrain. The advent of intersectional genetic strategies has allowed the unprecedented dissection of the roles of neurochemically-defined VP subtypes in motivated behavior. The discovery that VP_Glu_ neurons are distinct from canonical VP subtypes, and are critical for suppressing appetitive behavior, particularly under conditions of conflict, has made them a particularly exciting population to target in the context of addiction. We initiated this study as a first step towards understanding the molecular and biophysical determinants of VP_Glu_ neurons, which may ultimately be leveraged to modulate their activity for forward translation. However, while the distinction between “GABAergic” and “Glutamatergic” has been a useful starting point for dissecting VP function, our results call for caution when interpreting the effects of functional manipulations that affect the entire neurochemically-defined population.

Our patch clamp experiments revealed two distinct subpopulations of VP_Glu_ neurons: one population that entered depolarization block in response to large-amplitude current injections and one population that did not enter depolarization block (Fig 1d,e). Interestingly, the only differences between these subpopulations were in their firing properties; the passive membrane properties of these two populations were not different (Fig 1). As PV is a common marker for fast-spiking, depolarization block-insensitive interneurons throughout the nervous system and has been reported to functionally delineate VP subpopulations [21], we initially hypothesized that PV may be a marker for NDB VP_Glu_ neurons. However, this hypothesis was not supported by our modeling or RT-PCR results.

Simulating PV expression in our DB model population did not confer resistance to depolarization block (Fig 4c), although it did produce a leftward shift in the FI-curve and increased its peak amplitude, confirming that PV inclusion may increase firing in response to lower-amplitude current injections. Simulating low initial PV concentrations (1–2 mM), also shifted the NDB model population FI-curve to the left, producing an increased excitability in response to lower-amplitude current injections (Fig 4c). However, at higher concentrations (≥ 2 mM), PV caused the NDB population’s mean FI-curve to resemble a DB phenotype, i.e., an increase in firing rate with increased current injection amplitude until a peak firing current is reached followed by a decrease in firing rate in response to further increases in current injection amplitude. Our interpretation is that this PV-induced reduction in firing rate in response to larger-amplitude current injections is mediated by PV preventing the activation of SK channels. Calcium-activated SK channels would normally be triggered by calcium influx through voltage-gated calcium channels during the depolarization phase of an action potential. PV would serve to buffer a significant portion of this calcium inflow, thus preventing calcium-induced activation of SK. SK channels conventionally mediate the afterhyperpolarization phase of the action potential and thus modulate a neuron’s maximum firing frequency [56,57]. During the PV-induced reduction of SK activation, the neuron would not sufficiently repolarize following an action potential increasing the likelihood that sodium channels would remain in an inactivated state particularly in response to large-amplitude current injections.

Crucially, the intracellular concentration of PV in VP_Glu_ neurons is currently unknown, and PV protein expression is highly dynamic, influenced by recent cellular and synaptic activity [58,59]. We cannot determine PV protein expression from our single cell RT-PCR, which only allows us to categorize a VP_Glu_ neuron as PV-positive or PV-negative, based on gene expression. We therefore modeled intracellular PV concentrations between 1 and 10 mM and saw the most pronounced effects on excitability at concentrations ≥ 2 mM. In other neuronal populations, intracellular PV concentrations may be considerably lower than 1 mM [60]. Together, our results indicate that PV concentration in VP_Glu_ may be below the threshold to influence our measures of excitability, or more likely that PV is not the factor distinguishing electrophysiological populations of VP_Glu_ neurons. Other intracellular calcium buffering proteins, such as calbindin or calretinin, may be upregulated in DB VP_Glu_ neurons or serve as markers to differentiate NDB from DB neurons. While there is no existing evidence for differential calcium binding proteins in VP_Glu_ neurons, future experiments could test this hypothesis by quantifying expression of these proteins or examining the consequences of intracellular calcium chelators (e.g., EGTA, BAPTA) on the firing properties of DB and NDB VP_Glu_ neurons.

Inclusion of a PV mechanism in our model shifted the firing frequency and excitability as predicted (Fig 4). However, inclusion of a PV mechanism did not confer a fast-spiking phenotype, which was also evident in our electrophysiology data (Fig 5). While this is consistent with a prior study characterizing PV-positive VP neurons [21], this is seemingly in contrast to PV-positive neurons in the neocortex [59,61,62], hippocampus [22] or PV-positive glutamatergic neurons in the adjacent lateral hypothalamus [63], which all exhibit a fast-spiking phenotype. We hypothesize two reasons for these differences. First, the fast-spiking phenotype is associated with expression of the strong delayed rectifying, voltage-gated potassium channels Kv3.1, Kv3.2 and Kv1.6, encoded by the genes *Kcnc1, Kcnc2* and *Kcna6* respectively [63–65]. These genes were not expressed at detectable levels in our Ribotag data (Fig 2c) and were not incorporated in our models. Conversely, genes encoding the small-conductance potassium channel (SK) are negatively correlated with fast-spiking phenotypes [62], and the genes encoding the SK1 (*Kcnn1*) and SK2 (*Kcnn2*) channels were among the most abundantly expressed in our sequencing data, which is reflected in our model (Fig 2c,d). Second, PV-positive interneurons in the cortex express extensive dendritic arbors with excitatory synaptic contacts [66], and synaptic activity is a key source of calcium through calcium-permeable AMPA receptors, NMDA receptors, and dendritic calcium-activated or voltage-activated calcium channels. Our patch-clamp experiments were conducted in synaptic blockers, and our subsequent modeling did not incorporate synaptic input, which could also contribute to the absence of the fast-spiking phenotype in our PV-positive VP_Glu_ neurons. Even in the presence of synaptic blockers, electrotonic spread of somatic current injections to the dendrites could influence the activity of dendritic voltage-gated calcium channels. In future studies, these model populations could be extended to examine the influence of excitatory synaptic input, dendritic voltage-gated calcium channels, and their confluence with PV, on shaping the firing properties of VP_Glu_ neurons.

While our experimental results align well with our modeling results, there are limitations of the model that should be considered. Both the DB and NDB neuron model populations reproduced many electrophysiological properties of the experimental population (Fig 3b–f). However, these model populations may represent the less-excitable subsets of the experimental population (e.g., NDB neuron models skewing to the lower end of experimentally measured peak firing frequencies (Fig 3b,c,g). Notably, our model populations produced considerably lower input resistances than the experimental population, and the FI-curves show that the model populations underestimated the experimental population’s excitability in response to small current injection amplitudes (e.g., ≤ 40 pA). We believe that assumptions regarding our VP_Glu_ neuron model structure may contribute to these discrepancies. First, we represented the dendrite as a single cylindrical compartment, whereas VP_Glu_ neurons have a highly arborized dendritic structure. This structure would result in significantly higher overall dendritic impedances than our single compartment dendrite, which would likely recapitulate the higher input resistances measured experimentally (Fig 3 g). However, because the full cytoarchitecture of VP_Glu_ neurons is currently uncharacterized, our single-compartment approximation of the dendrite provides a valuable first investigation into the electrophysiological and firing properties of these neurons. When anatomical reconstructions of VP_Glu_ neurons become available, our modeling framework can be extended to account for such complexities and investigate the effect of realistic cell morphology on the electrophysiological characteristics of these cells. Second, our models contained active ion-channel conductances only in the axon hillock to reduce the dimensionality of parametrization with the MCMC algorithm and to improve computational efficiency. It is possible that including active ionic components in the somatic or dendritic compartments could further increase the excitability of our models, particularly in response to small current injection amplitudes. Future studies should incorporate the spatial distribution of active ionic conductances in VP_Glu_ neurons which could not be provided by transcriptomic data. Finally, we implemented PV calcium binding dynamics as detailed in the study of Bischop and colleagues on the firing of striatal fast-spiking neurons [45]. However, our implementation of the effects of PV on intracellular calcium dynamics did not account for PV binding to Mg^2+^. At rest, Mg^2+^ occupies the majority of PV Ca^2+^/Mg^2+^ binding sites [24]. We believe that our exclusion of modeling Mg^2+^ in our implementation of PV is a reasonable simplification because 1) PV has a significantly higher binding affinity for Ca^2+^ than Mg^2+^ [24], and 2) sensitivity analyses of DB and NDB model populations showed that reducing the association constant of PV with Ca^2+^ does not significantly affect the model population’s mean FI-curve (data not shown).

An open question is whether these DB or NDB subpopulations have distinct projection targets, which is important, given that projection target may confer these populations with unique functional roles in behavior [15,16,21,67–71]. Single cell reconstruction of individual VP neurons suggests that neurons project to either the habenula and midline thalamic targets or innervate the midbrain [72]. More contemporary studies have corroborated this early finding and established that non-overlapping populations of PV-positive VP neurons project to either the lateral habenula or to the midbrain, and at least a subset of both these PV-positive populations are glutamatergic [17,18,21]. Intriguingly, these anatomically distinct populations of PV-positive neurons exhibited differences in excitability in response to current injections, which may be consistent with the projection target (LHb or midbrain) accounting for differences in electrophysiological profiles. However, this study did not present a full intrinsic characterization of these neurons, nor did they distinguish between GABAergic or glutamatergic identity within the PV-positive population. To address the open question of whether DB and NDB populations align with unique anatomical projections, future studies would characterize firing properties of VP_Glu_ neurons while incorporating retrograde labeling to identify the neurons projection target.

## Conclusion

Glutamatergic neurons in the ventral pallidum (VP_Glu_) are a promising target for modulating reward seeking behavior. As a first step towards identifying rational strategies to modulate these neurons, we attempted to provide an electrophysiological and transcriptomic characterization. In this characterization, we found two electrophysiologically distinct subpopulations of VP_Glu_ neurons, which we hypothesized may align with PV-positive and -negative populations of VP neurons previously described. However, our modeling and experimental data did not support this hypothesis, finding that PV could not account for differences in these two populations. Future work will determine whether these biophysical populations correspond to distinct anatomical projection targets, and which ionic mechanisms can account for these biophysical differences.

Despite these limitations, our modeling approach, grounded in transcriptomic profiles, represents a significant step towards rationally modulating activity of these neurons. The expansion of this framework may allow for interrogation of mechanisms that drive changes in excitability of VP_Glu_ neurons in disease states, which is a necessary first step towards reversing these functional changes as a therapeutic strategy.

## Figures and Tables

**Table 1:** Voltage-gated ion channels and auxiliary subunits enriched in VP_Glu_ TRAP samples.

Gene name		Norm. Transcript Count
Kcnma1	potassium large conductance calcium-activated channel, subfamily M, alpha member 1	38
Cacna1c	calcium channel, voltage-dependent, L type, alpha 1C subunit	27
Kcnq2	Potassium Voltage-Gated Channel Subfamily Q Member 2	21
Scn8a	sodium channel, voltage-gated, type VIII, alpha	20
Cacna1g	calcium channel, voltage-dependent, T type, alpha 1 G subunit	16
Cacnb2	Calcium Voltage-Gated Channel Auxiliary Subunit Beta 2	16
Cbarp	CACN Subunit Beta Associated Regulatory Protein	15
Kcnt1	Potassium Sodium-Activated Channel Subfamily T Member 1	15
Kcnab2	Potassium voltage-gated channel, shaker-related subfamily, beta member 2	14
Kcnj15	Potassium Inwardly Rectifying Channel Subfamily J Member 15	13
Cacna1d	Calcium Voltage-Gated Channel Subunit Alpha1 D	12
Cacna2d1	calcium channel, voltage-dependent, alpha2/delta subunit 1	12
Clcn2	Chloride Voltage-Gated Channel 2	11
Cacna1f	Calcium Voltage-Gated Channel Subunit Alpha1 F	11
Cacna1a	Calcium Voltage-Gated Channel Subunit Alpha1 A	11
Clcn4	Chloride Voltage-Gated Channel 4	10
Cacna1b	calcium channel, voltage-dependent, N type, alpha 1B subunit	10
Clcn3	Chloride Voltage-Gated Channel 3	10
Cacna2d2	Calcium channel, voltage-dependent, alpha 2/delta subunit 2	10
Vdac2	Voltage Dependent Anion Channel 2	10
Cacna1h	Calcium Voltage-Gated Channel Subunit Alpha1 H	10
Clcn1	Chloride Voltage-Gated Channel 1	10
Kcnk2	Potassium Two Pore Domain Channel Subfamily K Member 2	10
Cacna2d4	Calcium channel, voltage-dependent, alpha 2/delta subunit 4	10
Kcnn2	Potassium Calcium-Activated Channel Subfamily N Member 2	10
Cacna2d3	Calcium Voltage-Gated Channel Auxiliary Subunit Alpha2delta 3	9
Cracr2b	Calcium Release Activated Channel Regulator 2B	9
Scn3b	Sodium Voltage-Gated Channel Beta Subunit 3	9
Scn2a	Sodium channel, voltage-gated, type II, alpha	9
Kcnn1	Potassium Calcium-Activated Channel Subfamily N Member 1	8

## Data Availability

RNA seq Data are available at the Gene Expression Omnibus under accession number: GSE272940. All other data available upon request.
